# Does Serum Uric Acid Mediate Relation between Healthy Lifestyle and Components of Metabolic Syndrome?

**DOI:** 10.3390/nu16132137

**Published:** 2024-07-04

**Authors:** Yan Huang, Hui Jing, Ziping Wang, Zongkai Li, Samuel Chacha, Yuxin Teng, Baibing Mi, Binyan Zhang, Yezhou Liu, Qiang Li, Yuan Shen, Jiaomei Yang, Yang Qu, Duolao Wang, Hong Yan, Shaonong Dang

**Affiliations:** 1 Department of Epidemiology and Biostatistics, School of Public Health, Xi’an Jiaotong University Health Science Center, Xi’an 710061, China; hyhuangyanhy@163.com (Y.H.); jingh3@stu.xjtu.edu.cn (H.J.); zipingw727@163.com (Z.W.); lzk2239522898@163.com (Z.L.); xjtu.mi@xjtu.edu.cn (B.M.); zhangbinyan@stu.xjtu.edu.cn (B.Z.); yezhouliu@stu.xjtu.edu.cn (Y.L.); tjlq@mail.xjtu.edu.cn (Q.L.); shenyuan_xjd@163.com (Y.S.); violetyjm18@xjtu.edu.cn (J.Y.); yanhonge@mail.xjtu.edu.cn (H.Y.); 2 Department of Molecular Diagnostics, Sumbwanga Regional Referral Hospital, Rukwa 413, Tanzania; chacha60351@gmail.com; 3 Department of Human Resources, The Second Affiliated Hospital of Xi’an Jiaotong University, Xi’an 710004, China; yx9966@stu.xjtu.edu.cn; 4 HKU Business School, 3/F K.K. Leung Building, The University of Hong Kong, Pokfulam Road, Hong Kong; charleneqy@163.com; 5 Department of Clinical Sciences, Liverpool School of Tropical Medicine, Liverpool L7 8XZ, UK; duolao.wang@liverpool.ac.uk; 6 Key Laboratory of Environment and Genes Related to Diseases, Xi’an Jiaotong University, Ministry of Education, Xi’an 710061, China

**Keywords:** healthy lifestyle, metabolic syndrome, uric acid, mediation analysis

## Abstract

A healthy lifestyle is related to metabolic syndrome (MetS), but the mechanism is not fully understood. This study aimed to examine the association of components of MetS with lifestyle in a Chinese population and potential mediation role of serum uric acid (SUA) in the association between lifestyle behaviors and risk of components of MetS. Data were derived from a baseline survey of the Shaanxi urban cohort in the Regional Ethnic Cohort Study in northwest China. The relationship between components of MetS, healthy lifestyle score (HLS), and SUA was investigated by logistic or linear regression. A counterfactual-based mediation analysis was performed to ascertain whether and to what extent SUA mediated the total effect of HLS on components of MetS. Compared to those with 1 or less low-risk lifestyle factors, participants with 4–5 factors had 43.6% lower risk of impaired glucose tolerance (OR = 0.564; 95%CI: 0.408~0.778), 60.8% reduction in risk of high blood pressure (OR = 0.392; 95%CI: 0.321~0.478), 69.4% reduction in risk of hypertriglyceridemia (OR = 0.306; 95%CI: 0.252~0.372), and 47.3% lower risk of low levels of HDL cholesterol (OR = 0.527; 95%CI: 0.434~0.641). SUA mediated 2.95% (95%CI: 1.81~6.16%) of the total effect of HLS on impaired glucose tolerance, 14.68% (95%CI: 12.04~18.85%) on high blood pressure, 17.29% (95%CI: 15.01~20.5%) on hypertriglyceridemia, and 12.83% (95%CI: 10.22~17.48%) on low levels of HDL cholesterol. Increased HLS tends to reduce risk of components of MetS partly by decreasing the SUA level, which could be an important mechanism by which lifestyle influences MetS.

## 1. Introduction

Combining risk factors for cardiovascular disease such as obesity, hyperglycemia, hypertension, and dyslipidemia, metabolic syndrome (MetS) has emerged as a global public health concern [1]. MetS has been shown to be a significant risk factor for diabetes [2], cardiovascular disease [3], and chronic kidney disease [4]. In developed nations, the prevalence of MetS has increased over the past ten years to approximately 25% of the adult population [5]. The standardized prevalence of MetS among Chinese aged 20 and older has risen to 31.1% [6]. However, it is expensive and time-consuming to perform widespread MetS screening using the current diagnostic standards. As a result, MetS control and prevention become more crucial.

Although the exact cause of MetS remains unknown, insulin resistance, oxidative stress, and chronic low-grade inflammation may play significant roles in its pathophysiology [7]. Lifestyle risk factors such as smoking, inactivity, and unhealthy eating patterns are considered major risk factors for MetS [8,9,10,11]. Investigating the health effects of a combination of lifestyle risk factors is important, as they are interrelated [12]. Furthermore, the cumulative impact of multiple lifestyle risk factors on health is greater than that of a single risk factor [13]. While these lifestyle factors could be involved in the occurrence and progression of MetS, the mechanism still remains unclear. Leading a healthy lifestyle appears to reduce the body’s levels of oxidative stress and inflammation, which are thought to be the typical pathological and physiological features of MetS [14,15,16,17].

Serum uric acid (SUA) is an enzymatic end product of purine metabolism in humans [18]. Various illnesses have been linked to changes in SUA homeostasis [19]. Although SUA levels are often associated with MetS [20], hyperuricemia is not included in the diagnostic criteria for this pathology. However, the pro-oxidative effect of hyperuricemia may induce inflammation and endothelial dysfunction by reducing the availability of nitric oxide, thereby promoting the development of MetS [19]. Meanwhile, a plethora of data indicate that people can dramatically lower their risk of hyperuricemia by leading healthy lifestyles [21,22,23,24]. Renal function-normalized SUA, the serum-uric-acid-to-creatinine ratio (SUA/Cr), is considered a more accurate representation of endogenous uric acid levels than SUA levels alone [25]. The uric-acid-to-HDL-cholesterol ratio (UHR) has been proposed as another novel inflammatory and metabolic marker [26]. However, it remains unclear to what extent SUA mediates the association between lifestyle behaviors and components of MetS. Therefore, to address this knowledge gap, using the data from a large-scale epidemiological survey in northwest China, the present study examined the potential association of components of MetS with combined lifestyle factors, including alcohol intake, smoking, body composition, physical activity, and dietary habits, in the Chinese population. Additionally, the potential mediation role of SUA in the association between lifestyle behaviors and the risk of components of MetS was explored.

## 2. Materials and Methods

### 2.1. Data and Participants

Data were derived from the baseline survey of the Shaanxi urban cohort in the Regional Ethnic Cohort Study in northwest China (RECS), and a detailed study design has been described elsewhere [27]. Participants who were permanent residents of the survey sites and aged between 20 and 89 years were included. Trained investigators conducted face-to-face interviews to collect the questionnaire data. For the present study, participants from the Shaanxi urban cohort (*n* = 13,072) were included. Those with missing data for lifestyle factors (*n* = 3065), SUA (*n* = 33), SUA/Cr (*n* = 2631), and UHR (*n* = 33), as well as an incomplete education level (*n* = 51) and marital status (*n* = 61), were excluded. As a result, a total of 7287 participants were included in the final analyses (Figure S1). The included and excluded populations differed slightly in terms of gender, marital status, lifestyle, and other demographic characteristics (Table S1).

### 2.2. Components of MetS

Four components of MetS were considered for the analysis and their diagnosis was based on the criteria outlined by the NCEP ATP III definition [28]. Specifically, the following criteria were used for each component: (1) impaired glucose tolerance—fasting plasma glucose ≥ 5.6 mmol/L or were using medications for type 2 diabetes mellitus; (2) high blood pressure—SBP ≥ 130 mmHg or DBP ≥ 85 mmHg or were using antihypertensive medications; (3) hypertriglyceridemia—triglycerides ≥ 1.7 mmol/L or were using lipid-lowering medications; and (4) low levels of HDL cholesterol—HDL < 1.3 mmol/L for women and <1 mmol/L for men. The component of abdominal obesity or overweight was not included because of its potential correlation with BMI, which is a part of HLS.

### 2.3. Healthy Lifestyle Score and Weighted HLS

A five-item healthy lifestyle score (HLS) was established, incorporating five lifestyle factors based on previous studies [29]. These factors included alcohol intake, smoking, body composition, physical activity, and dietary habits. For alcohol intake, participants who reported never drinking or moderate drinking were classified as low risk. Similarly, participants who reported never smoking or smoking occasionally were considered low risk for smoking. A low-risk body mass index (BMI) was defined as ranging from 18.5 to 23.9 kg/m^2^. The physical activity level was assessed based on calculated MET-h/day. Participants in the highest sex-specific quartile were categorized as having a high level of physical activity, which was considered low risk. For dietary habits, a dietary score ranging from 0 to 3 was estimated based on three food groups as fruits, vegetables, and red meat, which were selected based on previous evidence [29]. A healthy dietary habit was considered low risk and was defined as meeting at least two ideal diet components, classified as above the median intake of fruits and vegetables, and below the lowest quartile intake of red meat. Diet information was collected using a semi-quantitative food frequency questionnaire (FFQ). According to their eating habits over the previous 12 months, participants were asked to report the frequency and portion size of each food group they had consumed. The FFQ provided five options for each food group: “daily”, “4–6 times/week”, “1–3 times/week”, “1–3 times/month”, or “none or rarely”. These options were quantified as 7, 5, 2, 0.5, and 0 times per week, respectively, for the data analysis.

For each healthy lifestyle factor, the participants received a score of 1 if they met the criterion or 0 otherwise. Therefore, the total HLS score ranged from 0 (unhealthiest lifestyle) to 5 (healthiest lifestyle). Due to sparse data in the extreme scores, participants with scores 0 and 1 were combined, as were those with scores of 4 and 5, for further analyses. Considering the impact of each healthy lifestyle factor on the outcome, a weighted healthy lifestyle score (weighted HLS) was created as an alternative indicator for the sensitive analysis according to the formula as weighted HLS = (β1 × x1 + β2 × x2 + β3 × x3 + β4 × x4 + β5 × x5) × (5/sum of the β coefficients) [30]. The β coefficients were estimated using a logistic regression model with all five lifestyle factors as exposure variables and each component of MetS as the outcome (Table S2). In the analysis, each weighted HLS was divided into four categories according to their quartiles.

### 2.4. Biochemical and Physical Examination

A 10 mL fasting venous blood sample of the participant was collected by a qualified doctor or nurse at the cooperative unit for various biomarker tests. SUA and creatinine were measured using enzymatic or chromatographic methods. In addition to SUA, two other indicators were used. SUA/Cr was calculated by SUA (mmol/L)/Cr (mmol/L) [25] and UHR (%) was calculated by SUA (mg/dL)/HDL (mg/dL) × 100 [26]. SUA, SUA/Cr, and UHR were classified into four categories according to their quartiles. The physical examination included measurements of height, weight, and blood pressure. Height and weight were measured by a body composition meter (TANITA BC-567). Blood pressure was measured using medical arm electronic blood pressure monitors. BMI was calculated using the formula BMI (kg/m^2^) = weight (kg)/height (m^2^).

### 2.5. Mediating Conceptual Model and Covariates

Currently, the literature suggests a possible link between HLS, SUA, and MetS [13,19,29]. As a result, a conceptual model based on the counterfactual frame was hypothesized to depict a possible causal relationship between HLS and components of MetS via SUA, considering potential confounders, as shown in Figure S2. In this model, HLS was regarded as having not only a direct effect on components of MetS but also an indirect effect via SUA, which acts as a mediator. Meanwhile, some covariates influence HLS, SUA, or components of MetS, potentially confounding the above-mentioned relationship. Gender, age, education level, marital status, wealth index, unhealthy eating behaviors, staple foods, and history of major diseases (such as acute myocardial infarction, angina, stroke, and pulmonary heart disease) were considered covariates in this study [13,29,31]. The education level was categorized as middle school and below, and junior college and above. Marital status was categorized as married and others. Economic status was assessed using a wealth index, constructed through a principal component analysis, and the variables included family property (such as flush toilet, car, motorcycle/other motor vehicles, computers, internet system, and smart phone), building or newly renovated building within five years, and travel and vacation at own expense within five years. The wealth index score was constructed based on a principal component score and then divided into tertiles (low, medium, and high). Unhealthy eating behaviors were also assessed using a principal component analysis, with variables including frequency of snacking, convenience foods, late-night snacks, bacon, ham/sausages, fried foods, barbecues, fast foods, and frequency of skipping breakfast and eating out. These behaviors were deemed unhealthy due to their potential psychological and physical consequences. The unhealthy eating behaviors score was constructed based on a principal component score, and it was then divided into tertiles. Staple food intake was assessed based on the accumulated intake of rice, cooked wheat food, and cereals. A participant self-report was used to determine the history of acute myocardial infarction, angina, stroke, and pulmonary heart disease.

### 2.6. Statistical Analyses

Participants were classified into four groups according to HLS (0–1, 2, 3, 4–5) to present their characteristics. Continuous variables were analyzed using ANOVA and presented as the mean and standard deviation. Categorical variables were analyzed using the χ^2^ test and presented as frequencies and percentages. Logistic regression models were used to estimate odds ratios (ORs) and the 95% confidence interval (CI) for associations of components of MetS with HLS and weighted HLS, or SUA, SUA/Cr, and UHR, and the trend testing was performed by placing HLS and weighted HLS as continuous variables in the model. The models were adjusted for gender, age, education level, marital status, wealth index, unhealthy eating behaviors, staple foods, and history of major diseases. A multiple linear regression model was employed to assess the associations between HLS, weighted HLS, and SUA, SUA/Cr, and UHR. The exploratory joint effect of healthy lifestyle and SUA on components of MetS was evaluated and OR was estimated with significance set as 0.01 (99%CI) to compensate for the increase in type I error rates resulting from multiple testing. In the joint analysis, SUA, SUA/Cr, and UHR were classified to high and low categories according to their 75th percentile, respectively, and higher HLS was defined when a score was 3–5 points and higher weighted HLS was defined as scores higher than the 50th percentile. A counterfactual-based mediation analysis was conducted [32], using the medeff command in STATA, to ascertain whether and to what extent SUA, SUA/Cr, and UHR mediated the total effect of healthy lifestyle (HLS or weighted HLS) on components of MetS. The mediation analysis adjusted for the aforementioned covariates. Further, subgroup analyses were performed by gender (male or female) and age (<50 or ≥50 years) to investigate potential heterogeneity in the association of interest. To verify the robustness of the results, two sensitivity analyses were conducted with a repeating mediation analysis in the sample, excluding the participants with history of major disease (*n* = 7022), and in the imputed sample in which the missing variables were filled by the multiple imputation method (*n* = 13,072). Since HDL was used to calculate UHR, the relationship between low levels of HDL cholesterol and UHR was not examined. All analyses were performed using SPSS 26.0 and STATA 16.0 software. All tests were two-sided with *p* < 0.05 indicating statistical significance.

## 3. Results

### 3.1. Characteristics of Participants

The characteristics of the participants according to HLS are summarized in Table S3. Overall, the participants had a mean age of 41.98 years (SD: 13.42) and 49.2% of them were female. In total, 31% of participants had an ideal HLS (4–5 points), and 12.5% of them had a poor HLS (0–1 points).

### 3.2. Associations of the Components of MetS with HLS and Weighted HLS

The odds ratios of four components of MetS showed a significant decrease with an increasing HLS (Table 1). Compared to those with 1 or less low-risk lifestyle factors, participants with 4–5 factors had 43.6% lower odds of impaired glucose tolerance (OR = 0.564; 95%CI: 0.408~0.778), 60.8% reduction in the odds of high blood pressure (OR = 0.392; 95%CI: 0.321~0.478), 69.4% reduction in the odds of hypertriglyceridemia (OR = 0.306; 95%CI: 0.252~0.372), and 47.3% lower risk of low levels of HDL cholesterol (OR = 0.527; 95%CI: 0.434~0.641). When using weighted HLS instead of HLS, a similar tendency was observed.

In addition, the present study examined the association between each individual lifestyle factor and components of MetS and found that there was a significant association between components of MetS and alcohol intake, smoking, or body composition. However, it should be noted that healthy dietary habits and physical activity appeared to be related to lower odds of MetS components although there was no statistical significance (Table S4).

### 3.3. Associations of the Components of MetS with SUA, SUA/Cr, and UHR

The odds ratios of four components of MetS showed a significant increase with increasing levels of SUA, SUA/Cr, and UHR (Table 2). Compared to the lowest quartile, participants in the Q3 group had 44.3% higher odds of impaired glucose tolerance (OR = 1.443; 95%CI: 1.061~1.963). The odds of high blood pressure, hypertriglyceridemia, and low levels of HDL cholesterol in participants in the Q4 group were 2.594 (OR = 2.594; 95%CI: 2.129~3.161), 6.060 (OR = 6.060; 95%CI: 4.908~7.482), and 2.048 (OR = 2.048; 95%CI: 1.713~2.448) times higher than those in the Q1 group, respectively. Similar trends were observed when using SUA/Cr and UHR instead of SUA.

### 3.4. Associations between HLS, Weighted HLS, and SUA, SUA/Cr, and UHR

Participants with a healthier healthy lifestyle had significantly lower levels of SUA, SUA/Cr, and UHR regardless of using HLS or weighted HLS (Table S5).

### 3.5. The Joint Effect of HLS with SUA, SUA/Cr, and UHR on the Components of MetS

Lowering SUA levels and improving lifestyle have been shown to reduce the risk of developing components of MetS compared to individuals with higher SUA levels and unhealthy lifestyle (Table 3). A similar tendency was observed when weighted HLS was utilized instead of HLS (Table S6).

### 3.6. Role of SUA, SUA/Cr, and UHR Mediating Relationship between Healthy Lifestyle and the Components of MetS

**Figure 1:**
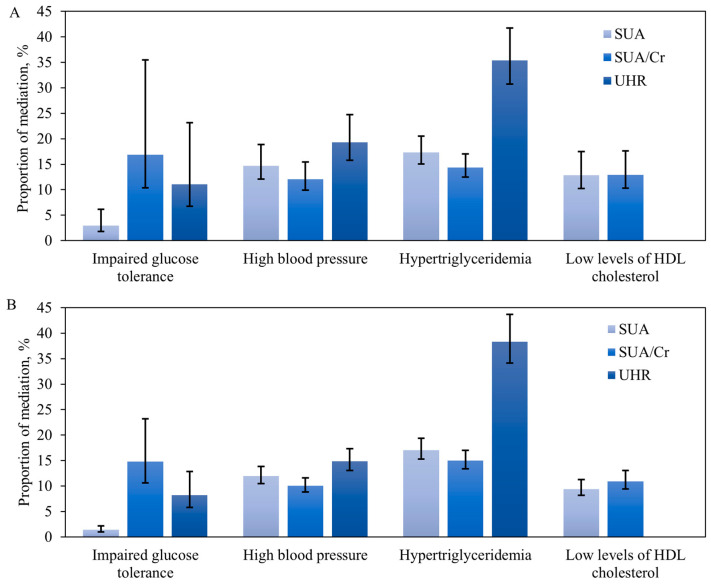
The mediation effect of SUA, SUA/Cr, or UHR on the path from HLS or weighted HLS to components of MetS. The mediation analysis was adjusted for gender, age, education level, marital status, wealth index, unhealthy eating behaviors, staple foods, acute myocardial infarction, angina, stroke, and pulmonary heart disease. (**A**) The association between HLS and components of MetS through SUA, SUA/Cr, or UHR. (**B**) The association between weighted HLS and components of MetS through SUA, SUA/Cr, or UHR. The bar represents the proportion of mediation, and the error line represents 95% CI. SUA: serum uric acid; SUA/Cr: serum-uric-acid-to-creatinine ratio; UHR: uric-acid-to-HDL-cholesterol ratio.

The impact of a healthy lifestyle on the components of MetS via SUA, SUA/Cr, and UHR was evaluated controlling for potential covariates by a mediation analysis (Figure 1A). SUA mediated 2.95% (95%CI: 1.81%~6.16%) of the total effect of HLS on impaired glucose tolerance, 14.68% (95%CI: 12.04%~18.85%) for high blood pressure, 17.29% (95%CI: 15.01%~20.5%) for hypertriglyceridemia, and 12.83% (95%CI: 10.22%~17.48%) for low levels of HDL cholesterol. SUA/Cr mediated 16.84% (95%CI: 10.38%~35.47%) of the total effect of HLS on impaired glucose tolerance, 12.07% (95%CI: 9.89%~15.47%) for high blood pressure, 14.36% (95%CI: 12.45%~17.04%) for hypertriglyceridemia, and 12.91% (95%CI: 10.30%~17.62%) for low levels of HDL cholesterol. UHR mediated 11.05% (95%CI: 6.74%~23.12%) of the total effect of HLS on impaired glucose tolerance, 19.27% (95%CI: 15.76%~24.74%) for high blood pressure, and 35.37% (95%CI: 30.71%~41.72%) for hypertriglyceridemia. When using weighted HLS as an alternative to HLS, a similar tendency was observed (Figure 1B).

### 3.7. Subgroup Analyses and Sensitivity Analyses

A stratified analysis based on gender and age was conducted to assess the heterogeneity in the mediation impact of SUA, SUA/Cr, or UHR on the association of lifestyle behaviors and components of MetS. Table S7 shows that there was a larger proportion mediated by SUA, SUA/Cr, or UHR among female participants than males regardless of components of MetS. Similarly, Table S8 shows that among participants aged over 50 years, there was a larger proportion mediated by SUA, SUA/Cr, or UHR. Furthermore, the mediation impact of SUA, SUA/Cr, or UHR remained consistent, and the mediated proportion did not significantly change when the mediation analysis was limited to participants without a history of major diseases. Additionally, the proportion mediated by SUA, SUA/Cr, or UHR did not significantly change when the mediation analysis was performed on all participants with imputed missing values (Table S9).

## 4. Discussion

The present study found that the HLS was inversely associated with risk of four components of MetS in the Chinese population. This association was partially mediated by the level of SUA. Compared with those with 1 or less low-risk lifestyle factors, the participants with 4–5 healthy factors had 43.6%, 60.8%, 69.4%, and 47.3% lower risk of impaired glucose tolerance, high blood pressure, hypertriglyceridemia, and low levels of HDL cholesterol, respectively. Similar results were observed when using weighted HLS instead of HLS.

Studies have shown that increased HLS can reduce the risk of MetS components [13,31]. Similar findings were found in the present study, but the effect size appeared to be lower, due to differences in the study population or the assessment of lifestyle. Moreover, an inverse association was observed for each individual healthy lifestyle factor; however, only alcohol intake, smoking, and body composition were significantly associated with reduced risk of components of MetS. This finding suggests that improving combined lifestyle behaviors against MetS is more crucial than focusing on a single behavior.

The exact relationship between HLS and MetS remains unknown; however, some studies suggest that smoking [9] and excessive alcohol consumption [8] can worsen blood lipid levels and increase insulin resistance. Physical activity has been shown to enhance metabolic status by improving fatty acid utilization, insulin sensitivity, and liver glucose production [11], while diet can modulate oxidative stress [10]. The joint analysis suggests that lowering SUA levels and improving lifestyle had a greater benefit in lowering the risk of developing components of MetS. Thereby, the present study proposed a potential mechanism by which SUA could mediate the relationship between a healthy lifestyle and components of MetS, implying that adopting a healthy lifestyle can help prevent or control risk of MetS by reducing SUA.

Uric acid is the final compound in the metabolic degradation of purine nucleotides [33]. It is produced in the liver. Purine nucleotides decompose to hypoxanthine and guanine, some of which can be recycled and phosphorylated into hypoxanthine nucleotides, while the remaining part is metabolized by a xanthine dehydrogenase/oxidase (XDH/XO) enzymatic reaction to the terminal product uric acid. XDH/XO is mainly expressed in parenchymal cells of the liver and small intestine. XDH has low reactivity and can be converted into XO. The production of uric acid mainly depends on the amount of substrate and the activity of XO. Finally, XDH/XO promotes the final step of purine metabolism, converting hypoxanthine to xanthine and converting xanthine to uric acid [34]. Changes in SUA concentration in human body fluids can reflect the metabolic status, immune system, and other human body functions. If the concentration of SUA in the blood exceeds the normal level, human body fluids will become acidic, thereby affecting the normal function of human cells and leading to long-term metabolic diseases [34]. SUA is associated with obesity, diabetes mellitus [34], hypertension, cardiovascular disease [35], and chronic kidney disease [36], in which SUA acts as an oxidant to induce oxidative stress and endothelial dysfunction [37]. Some indicators related to SUA were also found to be associated with MetS. SUA/Cr [25] and UHR [38] were found to be significantly correlated with the composition of MetS. The role of SUA in MetS and its pleiotropic effects on multiple organ systems have been a matter of discussion due to its complex and intricate connections within cellular metabolism and between signaling pathways [39]. A study has already focused on the effect of SUA on metabolism by illustrating its complex nature within the cellular metabolism and organ systems [40].

Raya-Cano et al. provided evidence that the concentration of SUA in subjects with MetS was significantly higher than in the control group by summarizing 42 studies, in which some Chinese studies were involved [19]. A cross-sectional study conducted in China found that the risk of MetS increased with the SUA level within the normal range, which supports the use of normal SUA levels as a clinical predictor of MetS, especially in women [41]. A survey of residents aged 60 and above in nine communities in northeast China showed a significant correlation between high SUA levels and MetS [42]. In another study based on rural Chinese, a positive correlation was found between SUA concentration and MetS, and this association was stronger in women than in men. And there was a negative association between SUA and hyperglycemia only in men [43]. The present study results were similar to those of previous studies, but the study population and effect size were different. Focusing on urban adults in northwest China, the effect size appeared larger than that of the aforementioned studies, which can provide corresponding measures for urban populations. Moreover, the putative association between SUA levels and diabetes mellitus is not clear. Previous studies have shown that there was a positive association between SUA and the risk of type 2 diabetes (T2DM), and other studies reported no association [44], while Pavani et al. [45] demonstrated that higher SUA concentration was inversely associated with T2DM. The present study found a negative correlation between SUA and impaired glucose tolerance in males, which is similar to the findings of Zhang et al. [43]. The inconsistent findings in the above studies may be explained by the differences in samples and criteria used to define MetS. Therefore, more studies of various populations are needed to investigate the SUA impact on hyperglycemia or diabetes mellitus.

The present mediation analyses revealed that SUA may be a potential pathway mechanism in the inverse association between combined lifestyle behavior and risk of MetS components. Previous studies have shown that SUA [29,46] and obesity [47] have a mediating role in the way from individual lifestyle factors to diabetes. The mediation mechanism of SUA between HLS and risk of MetS components may be associated with gut microbiota [48,49]. Interestingly, the present study found a negative correlation between SUA and impaired glucose tolerance in males but a positive correlation in females, which is similar to previous studies [50,51] and further caused a negative mediation impact of SUA and its related indicators in males. A possible explanation for such gender disparity was that low urate status may induce impaired glucose tolerance by increasing oxidative stress levels in males [50]. Importantly, the present study also found that the mediation effect of SUA, SUA/Cr, or UHR in the inverse association between HLS and risk of MetS components appeared to be greater among females and those aged above 50. This could be attributed to the fact that gut microbiota may affect material metabolism differently based on gender and age differences [52,53]. 

This study had several advantages. It comprehensively assessed the relationship between combined healthy lifestyles, SUA-related indicators, and components of MetS in a Chinese population. In addition, although the simple accumulation method has been widely used to calculate HLS, it may not account for the contribution of each lifestyle behavior to MetS. However, a weighted HLS was constructed based on the impact of each lifestyle factor on components of MetS. Nevertheless, there are several limitations that should be acknowledged. Firstly, the cross-sectional design used in this study did not allow for the establishment of an appropriate temporal sequence to demonstrate causality. Secondly, it was difficult to avoid recall bias in the collection of some covariates. Thirdly, although we have adjusted for some possible confounding factors, there were still some that cannot be captured, especially the exposure of the participants to environmental pollution, which could impact the health status of the participants and accessibility to medical services [54]. Lastly, the characteristics of the included and excluded populations were slightly different, which may limit the generalization of the results to some extent. However, these findings could be used as a reference to other populations with similar context to the present study.

## 5. Conclusions

A healthy lifestyle has the potential to decrease the risk of four components of MetS by reducing SUA. The significant mediating role of SUA could be an important mechanism by which lifestyle influences MetS. These findings imply that a healthy lifestyle could serve as an effective non-pharmacological approach with minimal side effects for maintaining a healthy metabolism in individuals. Additionally, management of individuals with elevated uric acid levels may be essential for the prevention and control of MetS.

## Figures and Tables

**Table 1:** Adjusted odd ratios for association of components of MetS with HLS and weighted HLS (*n* = 7287).

	Impaired Glucose Tolerance			High Blood Pressure			Hypertriglyceridemia			Low Levels of HDL Cholesterol		
	*n* (%)	OR (95%CI)	*p*	*n* (%)	OR (95%CI)	*p*	*n* (%)	OR (95%CI)	*p*	*n* (%)	OR (95%CI)	*p*
HLS												
0~1	100 (11.0)	Reference		444 (48.7)	Reference		471 (51.6)	Reference		281 (30.8)	Reference	
2	176 (10.8)	0.929 (0.705~1.225)	0.602	637 (39.0)	0.704 (0.590~0.840)	<0.001	642 (39.3)	0.702 (0.592~0.831)	<0.001	538 (32.9)	0.890 (0.744~1.065)	0.205
3	191 (7.7)	0.605 (0.453~0.807)	0.001	802 (32.3)	0.579 (0.484~0.692)	<0.001	645 (26.0)	0.453 (0.381~0.538)	<0.001	808 (32.6)	0.661 (0.551~0.792)	<0.001
4~5	131 (5.8)	0.564 (0.408~0.778)	<0.001	477 (21.1)	0.392 (0.321~0.478)	<0.001	377 (16.7)	0.306 (0.252~0.372)	<0.001	715 (31.6)	0.527 (0.434~0.641)	<0.001
		*P*_trend_	<0.001		*P*_trend_	<0.001		*P*_trend_	<0.001		*P*_trend_	<0.001
Weighted HLS												
Q1	281 (12.6)	Reference		883 (44.9)	Reference		1021 (47.4)	Reference		611 (37.0)	Reference	
Q2	138 (9.3)	0.807 (0.642~1.015)	0.067	672 (43.6)	0.884 (0.761~1.028)	0.110	518 (34.2)	0.704 (0.608~0.815)	<0.001	743 (36.3)	0.774 (0.672~0.891)	<0.001
Q3	108 (5.2)	0.494 (0.386~0.633)	<0.001	440 (23.0)	0.443 (0.380~0.516)	<0.001	319 (19.0)	0.326 (0.279~0.380)	<0.001	321 (25.1)	0.423 (0.357~0.502)	<0.001
Q4	71 (4.8)	0.481 (0.361~0.641)	<0.001	365 (19.6)	0.337 (0.285~0.398)	<0.001	277 (14.3)	0.289 (0.243~0.343)	<0.001	667 (28.8)	0.430 (0.369~0.501)	<0.001
		*P*_trend_	<0.001		*P*_trend_	<0.001		*P*_trend_	<0.001		*P*_trend_	<0.001

Note: Logistic regression model was used to estimate odds ratio (OR) and 95%CI with components of MetS as dependent variables and HLS/Weighted HLS as independent variable. Adjusted potential covariates included gender, age, education level, marital status, wealth index, unhealthy eating behaviors, staple foods, acute myocardial infarction, angina, stroke, and pulmonary heart disease. HLS: healthy lifestyle score.

**Table 2:** Adjusted odds ratios for association of the components of MetS with SUA, SUA/Cr, and UHR (*n* = 7287).

	*n*	Impaired Glucose Tolerance		High Blood Pressure		Hypertriglyceridemia		Low Levels of HDL Cholesterol	
		OR (95%CI)	*p*	OR (95%CI)	*p*	OR (95%CI)	*p*	OR (95%CI)	*p*
SUA									
Continuous variable		1.001 (0.999~1.002)	0.270	1.004 (1.004~1.005)	<0.001	1.007 (1.007~1.008)	<0.001	1.003 (1.002~1.004)	<0.001
Q1	1736	Reference		Reference		Reference		Reference	
Q2	1783	1.406 (1.039~1.904)	0.027	1.284 (1.071~1.541)	0.007	2.024 (1.658~2.471)	<0.001	1.203 (1.039~1.393)	0.013
Q3	1828	1.443 (1.061~1.963)	0.019	1.789 (1.483~2.158)	<0.001	3.203 (2.616~3.923)	<0.001	1.547 (1.316~1.819)	<0.001
Q4	1940	1.360 (0.988~1.871)	0.059	2.594 (2.129~3.161)	<0.001	6.060 (4.908~7.482)	<0.001	2.048 (1.713~2.448)	<0.001
SUA/Cr									
Continuous variable		1.284 (1.199~1.375)	<0.001	1.278 (1.221~1.337)	<0.001	1.496 (1.429~1.567)	<0.001	1.223 (1.174~1.275)	<0.001
Q1	1794	Reference		Reference		Reference		Reference	
Q2	1832	1.060 (0.808~1.390)	0.674	1.134 (0.962~1.337)	0.133	1.444 (1.216~1.715)	<0.001	1.217 (1.049~1.410)	0.009
Q3	1827	1.110 (0.848~1.453)	0.446	1.553 (1.322~1.824)	<0.001	2.313 (1.961~2.729)	<0.001	1.398 (1.208~1.619)	<0.001
Q4	1834	1.984 (1.546~2.546)	<0.001	2.101 (1.792~2.464)	<0.001	3.628 (3.082~4.270)	<0.001	1.883 (1.630~2.175)	<0.001
UHR									
Continuous variable		1.035 (1.015~1.056)	0.001	1.086 (1.072~1.101)	<0.001	1.262 (1.241~1.283)	<0.001	—	—
Q1	1701	Reference		Reference		Reference		—	
Q2	1854	1.451 (1.052~2.001)	0.023	1.485 (1.234~1.788)	<0.001	3.749 (2.923~4.809)	<0.001	—	—
Q3	1839	1.904 (1.380~2.626)	<0.001	2.125 (1.751~2.579)	<0.001	8.668 (6.739~11.148)	<0.001	—	—
Q4	1893	1.850 (1.317~2.598)	<0.001	3.123 (2.542~3.838)	<0.001	25.137 (19.317~32.711)	<0.001	—	—

Note: Logistic regression model was used to estimate odds ratio (OR) and 95%CI with components of MetS as dependent variables and SUA, SUA/Cr and UHR as independent variables. Adjusted potential covariates included gender, age, education level, marital status, wealth index, unhealthy eating behaviors, staple foods, acute myocardial infarction, angina, stroke, pulmonary heart disease, and HLS. SUA: serum uric acid; SUA/Cr: serum-uric-acid-to-creatinine ratio; UHR: uric-acid-to-HDL-cholesterol ratio. “—” means that relationship between low levels of HDL cholesterol and UHR was not examined since HDL was used to calculate UHR.

**Table 3:** The joint effect of HLS with SUA, SUA/Cr, and UHR on components of MetS (*n* = 7287).

	HLS	*n*	Impaired Glucose Tolerance		High Blood Pressure		Hypertriglyceridemia		Low Levels of HDL Cholesterol	
			OR (99%CI)	*p*	OR (99%CI)	*p*	OR (99%CI)	*p*	OR (99%CI)	*p*
SUA										
Higher	Lower	1072	Reference		Reference		Reference		Reference	
Higher	Higher	747	0.672 (0.426~1.061)	0.025	0.670 (0.511~0.879)	<0.001	0.581 (0.449~0.752)	<0.001	0.722 (0.549~0.948)	0.002
Lower	Lower	1473	1.127 (0.787~1.612)	0.391	0.545 (0.433~0.686)	<0.001	0.412 (0.330~0.514)	<0.001	0.688 (0.546~0.866)	<0.001
Lower	Higher	3995	0.669 (0.461~0.970)	0.005	0.367 (0.291~0.463)	<0.001	0.210 (0.167~0.264)	<0.001	0.455 (0.360~0.574)	<0.001
SUA/SCr										
Higher	Lower	752	Reference		Reference		Reference		Reference	
Higher	Higher	1069	0.698 (0.456~1.068)	0.029	0.594 (0.446~0.790)	<0.001	0.562 (0.428~0.738)	<0.001	0.733 (0.560~0.959)	0.003
Lower	Lower	1793	0.562 (0.391~0.808)	<0.001	0.522 (0.410~0.666)	<0.001	0.441 (0.349~0.558)	<0.001	0.672 (0.529~0.853)	<0.001
Lower	Higher	3673	0.355 (0.246~0.512)	<0.001	0.362 (0.284~0.461)	<0.001	0.226 (0.179~0.286)	<0.001	0.444 (0.351~0.561)	<0.001
UHR										
Higher	Lower	1094	Reference		Reference		Reference		—	
Higher	Higher	727	0.790 (0.513~1.218)	0.161	0.664 (0.506~0.871)	<0.001	0.623 (0.481~0.807)	<0.001	—	—
Lower	Lower	1451	1.034 (0.725~1.475)	0.808	0.550 (0.437~0.691)	<0.001	0.236 (0.188~0.297)	<0.001	—	—
Lower	Higher	4015	0.575 (0.396~0.835)	<0.001	0.374 (0.297~0.472)	<0.001	0.121 (0.096~0.154)	<0.001	—	—

Note: Higher SUA, SUA/Cr, and UHR were defined as scores higher than the 75th percentile; higher HLS was defined as 3–5. The logistic regression model was used to estimate the odds ratio (OR) and 99%CI. The adjusted potential covariates included gender, age, education level, marital status, wealth index, unhealthy eating behaviors, staple foods, acute myocardial infarction, angina, stroke, and pulmonary heart disease. HLS: healthy lifestyle score; SUA: serum uric acid; SUA/Cr: serum-uric-acid-to-creatinine ratio; UHR: uric-acid-to-HDL-cholesterol ratio. “—” means that the relationship between low levels of HDL cholesterol and UHR was not examined since HDL was used to calculate UHR.

## Data Availability

The datasets are not available for download to protect the confidentiality of the participants. The data are held at the School of Public Health, Xi’an Jiaotong University Health Science Center.

## Supplementary Materials

The following supporting information can be downloaded at: https://www.mdpi.com/article/10.3390/nu16132137/s1, Figure S1: The flow diagram of the participants included in the present study; Figure S2: Conceptual model of association between HLS and components of MetS via SUA considering potential confounders; Table S1: The characteristics of the participants included and excluded; Table S2: β coefficients of healthy lifestyle factors for calculating weighted healthy lifestyle score; Table S3: The characteristics of the participants according to HLS; Table S4: Adjusted odd ratios for association of components of MetS with healthy lifestyle factors in the model; Table S5: Adjusted β coefficient for association of HLS and weighted HLS with SUA, SUA/Cr, and UHR from liner regression model; Table S6: The joint effect of weighted HLS with SUA, SUA/Cr, and UHR on components of MetS; Table S7: Mediation impact of SUA, SUA/Cr, or UHR on the association between HLS or weighted HLS and components of MetS stratified by gender; Table S8: Mediation impact of SUA, SUA/Cr, or UHR on the association between HLS or weighted HLS and components of MetS stratified by age; Table S9: Mediation impact of SUA, SUA/Cr, or UHR on the association between HLS or weighted HLS and components of MetS among the participants without diseases or the sample with multiple imputation.

## Abbreviations

MetS: metabolic syndrome; SUA: serum uric acid; SUA/Cr: serum-uric-acid-to-creatinine ratio; UHR: uric-acid-to-HDL-cholesterol ratio; HLS: healthy lifestyle score; weighted HLS: weighted healthy lifestyle score; OR: odds ratio; CI: confidence interval.
