# Supplementary material for: Does Serum Uric Acid Mediate Relation between Healthy Lifestyle and Components of Metabolic Syndrome?
Source: Nutrients. 2024 Jul 4;16(13):2137. doi: 10.3390/nu16132137 (PMC11243389; doi:10.3390/nu16132137)
Supplement: Supplementary file 1 [file nutrients-16-02137-s001.zip › nutrients-3048210-supplementary.pdf]

Supplementary documents

Does Serum Uric Acid Mediate Relation between Healthy Lifestyle and Components of Metabolic Syndrome?

Contents

Figure S1. Flow diagram of the participants included in the present study

Figure S2. Conceptual model of association between HLS and components of MetS via SUA considering potential confounders

Table S1. The characteristics of the participants included and excluded

Table S2. β coefficients of healthy lifestyle factors for calculating weighted healthy lifestyle score

Table S3. The characteristics of the participants according to HLS

Table S4. Adjusted odd ratios for association of components of MetS with healthy lifestyle factors in the model

Table S5. Adjusted β coefficient for association of HLS and weighted HLS with SUA, SUA/Cr and UHR from liner regression model

Table S6. The joint effect of weighted HLS with SUA, SUA/Cr, and UHR on components of MetS

Table S7. Mediation impact of SUA, SUA/Cr or UHR on the association between HLS or weighted HLS and components of MetS stratified by gender

Table S8. Mediation impact of SUA, SUA/Cr or UHR on the association between HLS or weighted HLS and components of MetS stratified by age

Table S9. Mediation impact of SUA, SUA/Cr or UHR on the association between HLS or weighted HLS and components of MetS among the participants without diseases or the sample with multiple imputation

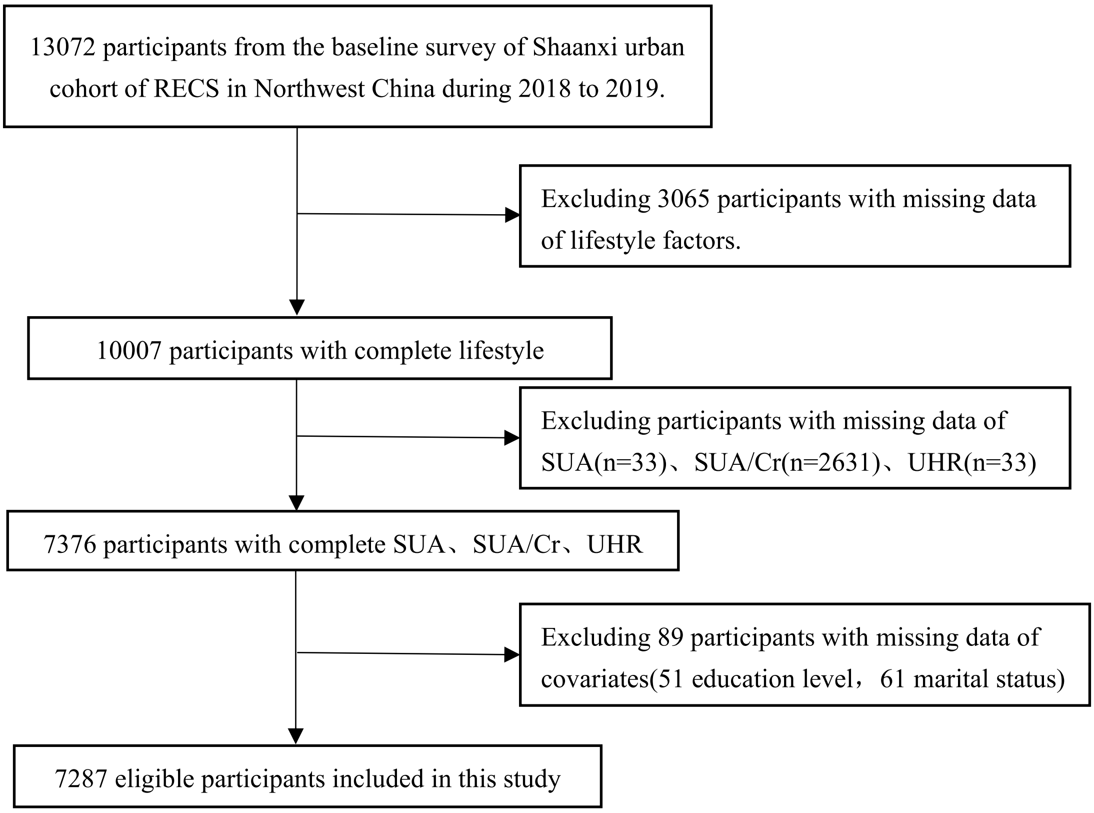

Figure S1. Flow diagram of the participants included in the present study

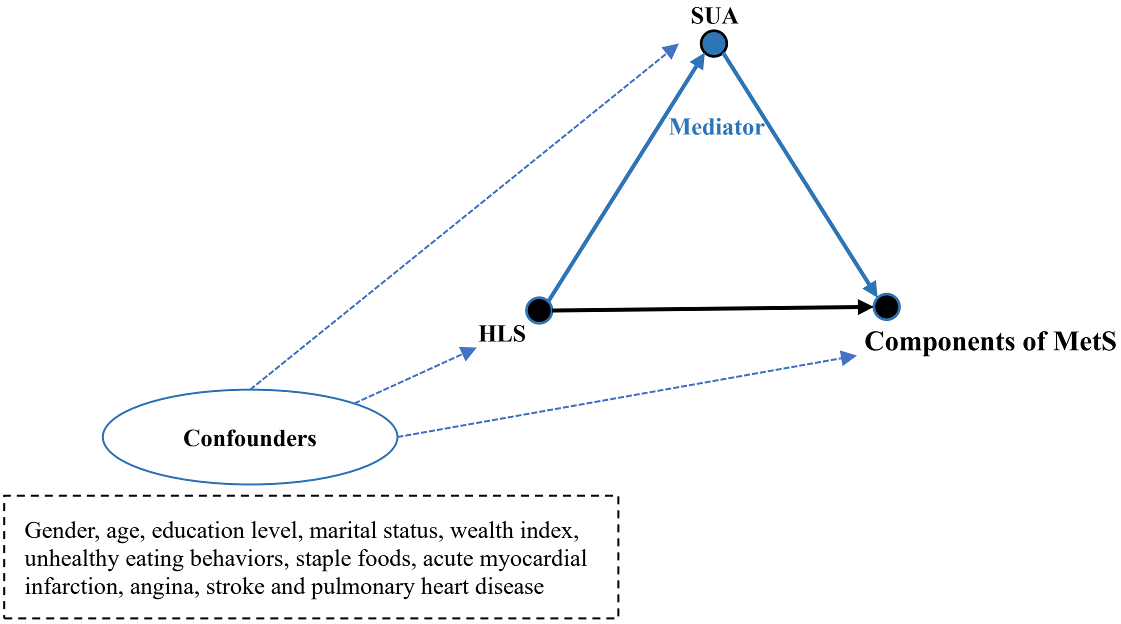

Figure S2. Conceptual model of association between HLS and components of MetS via SUA considering potential confounders

Table S1. The characteristics of the participants included and excluded

Variables	Inclusion	Exclusion	t/χ^2	*P*
N	7287	5785		
Gender			22.422	<0.001
Male	3701(50.8)	3179(55.0)		
Female	3586(49.2)	2606(45.0)		
Age(years)	41.98±13.42	38.86±13.05	178.251	<0.001
Education level			0.328	0.567
Middle school and below	854(11.7)	640(11.4)		
Junior college and above	6433(88.3)	4977(88.6)		
Married	5994(82.3)	4263(76.2)	71.082	<0.001
Wealth index			100.733	<0.001
Low	2553(35.0)	2522(43.6)		
Median	2051(28.1)	1455(25.2)		
High	2683(36.8)	1808(31.3)		
Unhealthy eating behaviors			94.192	<0.001
Healthy	2224(30.5)	2110(36.5)		
Median	2690(36.9)	1691(29.2)		
Unhealthy	2373(32.6)	1984(34.3)		
Staple foods(g/day）	194.05±131.94	208.76±154.16	34.489	<0.001
History of disease				
Acute myocardial infarction	54(0.7)	34(0.6)	1.134	0.287
Angina	110(1.5)	65(1.1)	3.637	0.057
Stroke	94(1.3)	50(0.9)	5.363	0.021
Pulmonary heart disease	41(0.6)	26(0.4)	0.811	0.368
Healthy lifestyle factors				
Never/moderate drinking	5769(79.2)	4335(78.2)	1.640	0.200
Never smoking /smoking occasionally	5975(82.0)	4289(79.0)	17.914	<0.001
Low-risk BMI	3778(51.8)	2932(50.7)	1.746	0.186
High physical activity level	1750(24.0)	1262(26.5)	9.372	0.002
Healthy dietary habit	3598(49.4)	1700(47.8)	2.479	0.115
Components of MetS				
Impaired glucose tolerance	598(8.2)	559(9.7)	8.480	0.004
High blood pressure	2360(32.4)	2106(36.4)	23.148	<0.001
Hypertriglyceridemia	2135(29.3)	1574(27.2)	6.934	0.008
Low levels of HDL cholesterol	2342(32.1)	1721(29.7)	8.600	0.003
SUA(μmol/L)	330.86±88.69	321.24±88.1	37.995	<0.001
SUA/Cr	5.40±1.22	5.38±1.26	0.478	0.489
UHR(%)	12.05±4.96	11.71±5.08	14.974	<0.001

Note: Participants excluded have incomplete value for education level(*n*=5617), marital status(*n*=5593), drinking(*n*=5541), smoking(*n*=5429), physical activity level(*n*=4765), dietary habit(*n*=3559), SUA(*n*=5743), SUA/Cr(*n*=2430), UHR(*n*=5743)

Table S2. β coefficients of healthy lifestyle factors for calculating weighted healthy lifestyle score

Healthy lifestyle factor	β coefficient			
	Impaired glucose tolerance	High blood pressure	Hypertriglyceridemia	Low levels of HDL cholesterol
Never/moderate drinking	-0.248	-0.251	-0.174	0.235
Never smoking /smoking occasionally	-0.003	0.148	-0.388	-0.345
Low-risk BMI	-0.656	-0.881	-0.981	-0.696
High physical activity level	0.017	-0.026	-0.084	-0.054
Healthy dietary habit	0.061	-0.096	-0.080	-0.007

Table S3. The characteristics of the participants according to HLS

Variables	Overall	HLS				F/χ^2	P
		0-1	2	3	4-5		
N	7287	912	1633	2480	2262		
Gender						1953.846	<0.001
Male	3701(50.8)	887(97.3)	1222(74.8)	1091(44.0)	501(22.1)		
Female	3586(49.2)	25(2.7)	411(25.2)	1389(56.0)	1761(77.9)		
Age(years)	41.98±13.42	40.43±10.9	41.5±13.03	42.99±14.48	41.83±13.35	9.586	<0.001
Education level						14.641	0.002
Middle school and below	854(11.7)	77(8.4)	181(11.1)	324(13.1)	272(12.0)		
Junior college and above	6433(88.3)	835(91.6)	1452(88.9)	2156(86.9)	1990(88.0)		
Married	5994(82.3)	781(85.6)	1338(81.9)	1985(80)	1890(83.6)	18.208	<0.001
Wealth index						18.123	0.006
Low	2553(35.0)	288(31.6)	584(35.8)	868(35.0)	813(35.9)		
Median	2051(28.1)	239(26.2)	434(26.6)	732(29.5)	646(28.6)		
High	2683(36.8)	385(42.2)	615(37.7)	880(35.5)	803(35.5)		
Unhealthy eating behaviors						68.926	<0.001
Healthy	2224(30.5)	207(22.7)	469(28.7)	819(33.0)	729(32.2)		
Median	2690(36.9)	342(37.5)	590(36.1)	858(34.6)	900(39.8)		
Unhealthy	2373(32.6)	363(39.8)	574(35.2)	803(32.4)	633(28.0)		
Acute myocardial infarction	54(0.7)	6(0.7)	13(0.8)	23(0.9)	12(0.5)	2.687	0.442
Angina	110(1.5)	8(0.9)	25(1.5)	46(1.9)	31(1.4)	4.741	0.192
Stroke	94(1.3)	6(0.7)	27(1.7)	40(1.6)	21(0.9)	8.909	0.031
Pulmonary heart disease	41(0.6)	6(0.7)	14(0.9)	14(0.6)	7(0.3)	5.274	0.153
Staple foods(g/day)	194.05±131.94	195.65±101.57	189.4±105.67	191.54±122.58	199.52±165.37	2.316	0.074
Healthy lifestyle factors							

Never/moderate drinking	5769(79.2)	170(18.6)	1105(67.7)	2266(91.4)	2228(98.5)	2893.276	<0.001
Never smoking /smoking occasionally	5975(82.0)	231(25.3)	1219(74.6)	2284(92.1)	2241(99.1)	2661.600	<0.001
Low-risk BMI	3778(51.8)	109(12.0)	366(22.4)	1308(52.7)	1995(88.2)	2346.021	<0.001
High physical activity level	1750(24.0)	64(7.0)	193(11.8)	424(17.1)	1069(47.3)	1012.288	<0.001
Healthy dietary habit	3598(49.4)	125(13.7)	383(23.5)	1158(46.7)	1932(85.4)	2085.449	<0.001
Components of MetS							
Impaired glucose tolerance	598(8.2)	100(11.0)	176(10.8)	191(7.7)	131(5.8)	41.898	<0.001
High blood pressure	2360(32.4)	444(48.7)	637(39.0)	802(32.3)	477(21.1)	275.201	<0.001
Hypertriglyceridemia	2135(29.3)	471(51.6)	642(39.3)	645(26.0)	377(16.7)	486.134	<0.001
Low levels of HDL cholesterol	2342(32.1)	281(30.8)	538(32.9)	808(32.6)	715(31.6)	1.737	0.629
SUA (μmol/L)	330.86±88.69	392.29±76.15	362.17±88.61	324.36±86.59	290.62±72.31	440.950	<0.001
SUA/Cr	5.40±1.22	5.62±1.20	5.52±1.28	5.38±1.22	5.25±1.17	26.618	<0.001
UHR (%)	12.05±4.96	15.74±4.72	13.89±5.13	11.62±4.68	9.71±3.72	502.266	<0.001

HLS: healthy lifestyle score; SUA: serum uric acid; SUA/Cr: serum uric acid to creatinine ratio; UHR: uric acid to HDL cholesterol ratio.

Table S4. Adjusted odd ratios for association of components of MetS with healthy lifestyle factors in the model (*n*=7287)

Healthy lifestyle factor	Impaired glucose tolerance		High blood pressure		Hypertriglyceridemia		Low levels of HDL cholesterol	
	OR (95%CI)	*P*	OR (95%CI)	*P*	OR (95%CI)	*P*	OR (95%CI)	*P*
Alcohol intake								
Unhealthy	Reference		Reference		Reference		Reference	
Healthy	0.780(0.623~0.977)	0.031	0.778(0.675~0.896)	0.001	0.840(0.732~0.964)	0.013	1.265(1.093~1.463)	0.002
Smoking								
Unhealthy	Reference		Reference		Reference		Reference	
Healthy	0.997(0.786~1.265)	0.981	1.160(0.997~1.349)	0.054	0.678(0.587~0.783)	<0.001	0.708(0.607~0.827)	<0.001
Body composition								
Unhealthy	Reference		Reference		Reference		Reference	
Healthy	0.519(0.429~0.628)	<0.001	0.414(0.369~0.465)	<0.001	0.375(0.335~0.420)	<0.001	0.498(0.447~0.555)	<0.001
Physical activity								
Unhealthy	Reference		Reference		Reference		Reference	
Healthy	1.017(0.810~1.276)	0.887	0.975(0.853~1.114)	0.707	0.919(0.806~1.049)	0.211	0.948(0.840~1.069)	0.380
Dietary habit								
Unhealthy	Reference		Reference		Reference		Reference	
Healthy	1.063(0.882~1.281)	0.523	0.909(0.808~1.022)	0.109	0.923(0.823~1.036)	0.173	0.993(0.893~1.104)	0.890

Note: Logistic regression model was used to estimate odds ratio (OR) and 95% CI. The adjusted potential covariates included gender, age, education level, marital status, wealth index, unhealthy eating behaviors, staple foods, acute myocardial infarction, angina, stroke, pulmonary heart disease.

Table S5. Adjusted β coefficient for association of HLS and weighted HLS with SUA, SUA/Cr and UHR from liner regression model (*n*=7287)

	SUA (μmol/L)		SUA/Cr		UHR(%)	
	β (95%CI)	*P*	β (95%CI)	*P*	β (95%CI)	*P*
HLS	-9.043(-10.686~-7.400)	<0.001	-0.127(-0.155~-0.099)	<0.001	-0.621(-0.712~-0.529)	<0.001
Weighted $HLS_{\text{Impaired glucose tolerance}}$	-6.716(-7.524~-5.909)	<0.001	-0.105(-0.119~-0.091)	<0.001	-0.470(-0.515~-0.426)	<0.001
Weighted $HLS_{\text{High blood pressure}}$	-7.041(-7.856~-6.225)	<0.001	-0.107(-0.121~-0.093)	<0.001	-0.480(-0.525~-0.434)	<0.001
Weighted $HLS_{\text{Hypertriglyceridemia}}$	-8.907(-10.000~-7.814)	<0.001	-0.144(-0.163~-0.125)	<0.001	-0.670(-0.730~-0.610)	<0.001
Weighted $HLS_{\text{Low levels of HDL cholesterol}}$	-5.764(-6.542~-4.986)	<0.001	-0.099(-0.113~-0.086)	<0.001	-0.474(-0.517~-0.432)	<0.001

Note: Liner regression model was used to estimate odds ratio (OR) and 95% CI. The adjusted potential covariates included gender, age, education level, marital status, wealth index, unhealthy eating behaviors, staple foods, acute myocardial infarction, angina, stroke, pulmonary heart disease. HLS: healthy lifestyle score; SUA: serum uric acid; SUA/Cr: serum uric acid to creatinine ratio; UHR: uric acid to HDL cholesterol ratio.

Table S6. The joint effect of weighted HLS with SUA, SUA/Cr, and UHR on components of MetS (*n*=7287)

	Weighted HLS	Impaired glucose tolerance		High blood pressure		Hypertriglyceridemia		Low levels of HDL cholesterol	
		OR (99%CI)	*P*	OR (99%CI)	*P*	OR (99%CI)	*P*	OR (99%CI)	*P*
SUA									
Higher	Lower	Reference		Reference		Reference		Reference	
Higher	Higher	0.594(0.340~1.036)	0.016	0.337(0.248~0.459)	<0.001	0.363(0.269~0.491)	<0.001	0.492(0.355~0.681)	<0.001
Lower	Lower	1.168(0.859~1.587)	0.193	0.545(0.443~0.671)	<0.001	0.410(0.337~0.498)	<0.001	0.695(0.569~0.849)	<0.001
Lower	Higher	0.586(0.411~0.836)	<0.001	0.263(0.212~0.328)	<0.001	0.170(0.137~0.211)	<0.001	0.365(0.294~0.453)	<0.001
SUA/SCr									
Higher	Lower	Reference		Reference		Reference		Reference	
Higher	Higher	0.564(0.352~0.904)	0.002	0.387(0.287~0.523)	<0.001	0.409(0.307~0.545)	<0.001	0.552(0.422~0.724)	<0.001
Lower	Lower	0.556(0.413~0.747)	<0.001	0.594(0.484~0.729)	<0.001	0.465(0.383~0.564)	<0.001	0.693(0.571~0.840)	<0.001
Lower	Higher	0.318(0.227~0.446)	<0.001	0.273(0.221~0.338)	<0.001	0.183(0.148~0.226)	<0.001	0.359(0.294~0.438)	<0.001
UHR									
Higher	Lower	Reference		Reference		Reference		—	
Higher	Higher	0.903(0.544~1.499)	0.604	0.296(0.214~0.411)	<0.001	0.426(0.316~0.576)	<0.001	—	—
Lower	Lower	1.109(0.816~1.508)	0.384	0.552(0.448~0.679)	<0.001	0.237(0.194~0.290)	<0.001	—	—
Lower	Higher	0.502(0.350~0.719)	<0.001	0.278(0.225~0.345)	<0.001	0.104(0.083~0.131)	<0.001	—	—

Note: Higher SUA, SUA/Cr and UHR were defined as scores higher than the 75th percentile; Higher weighted HLS was defined as scores higher than the 50th percentile. Logistic regression model was used to estimate odds ratio (OR) and 99% CI. The adjusted potential covariates included gender, age, education level, marital status, wealth index, unhealthy eating behaviors, staple foods, acute myocardial infarction, angina, stroke, pulmonary heart disease. HLS: healthy lifestyle score; SUA: serum uric acid; SUA/Cr: serum uric acid to creatinine ratio; UHR: uric acid to HDL cholesterol ratio. "—" means that the relationship between low levels of HDL cholesterol and UHR was not examined since HDL was used to calculate UHR.

Table S7. Mediation impact of SUA, SUA/Cr or UHR on the association between HLS or weighted HLS and components of MetS stratified by gender

	Male (*n*=3701)			Female (*n*=3586)		
	SUA	SUA/Cr	UHR	SUA	SUA/Cr	UHR
HLS						
Impaired glucose tolerance	-7.10(-23.90~-3.90)	12.51(6.86~38.66)	6.58(3.60~21.38)	12.98(5.62~63.38)	20.43(8.44~109.96)	15.01(6.29~77.15)
High blood pressure	12.28(9.95~16.27)	10.91(8.85~14.49)	17.91(14.50~23.73)	13.62(8.99~26.21)	12.93(8.51~25.19)	16.86(10.98~33.25)
Hypertriglyceridemia	14.56(12.54~17.66)	12.63(10.87~15.31)	33.89(29.16~40.61)	17.68(12.87~28.05)	16.92(12.21~27.46)	36.89(25.65~61.99)
Low levels of HDL cholesterol	7.95(5.96~11.97)	6.70(5.02~10.11)	—	18.87(12.87~38.99)	21.41(14.54~44.34)	—
Weighted HLS						
Impaired glucose tolerance	-8.97(-17.11~-5.80)	9.66(6.35~17.63)	3.18(2.08~5.94)	11.94(6.63~29.36)	22.44(12.61~56.17)	14.68(8.10~37.19)
High blood pressure	9.73(8.59~11.31)	8.11(7.16~9.43)	12.67(11.17~14.68)	11.62(8.68~17.31)	12.69(9.45~18.97)	16.45(12.19~24.65)
Hypertriglyceridemia	15.04(13.43~17.15)	12.71(11.36~14.51)	37.98(33.92~43.23)	16.74(13.11~22.82)	18.10(14.11~24.77)	38.71(29.71~53.83)
Low levels of HDL cholesterol	4.66(3.91~5.86)	3.93(3.29~4.94)	—	14.80(11.45~21.40)	19.57(15.12~28.37)	—

Note: SUA、SUA/Cr and UHR were included in the analysis as continuous variables, HLS and weighted HLS were included in the analysis as categorical variables; Models were adjusted for age, education level, marital status, wealth index, unhealthy eating behaviors, staple foods, acute myocardial infarction, angina, stroke, pulmonary heart disease. HLS: healthy lifestyle score; SUA: serum uric acid; SUA/Cr: serum uric acid to creatinine ratio; UHR: uric acid to HDL cholesterol ratio. "—" means that the relationship between low levels of HDL cholesterol and UHR was not examined since HDL was used to calculate UHR.

Table S8. Mediation impact of SUA, SUA/Cr or UHR on the association between HLS or weighted HLS and components of MetS stratified by age.

	<50 years (*n*=5415)			≥50 years (*n*=1872)		
	SUA	SUA/Cr	UHR	SUA	SUA/Cr	UHR
HLS						
Impaired glucose tolerance	2.87(1.52~7.22)	13.46(7.18~34.43)	12.36(6.50~31.53)	5.55(-47.36~79.40)	22.55(-171.94~461.48)	11.87(-104.91~175.57)
High blood pressure	11.81(9.18~16.48)	11.17(8.70~15.58)	15.61(12.08~21.72)	15.13(11.32~26.99)	8.65(6.49~15.46)	23.56(17.58~41.94)
Hypertriglyceridemia	15.87(13.37~19.81)	13.48(11.33~16.88)	33.13(27.86~41.17)	16.09(12.14~25.50)	14.44(10.87~23.02)	39.77(30.14~62.04)
Low levels of HDL cholesterol	13.61(10.22~20.70)	14.59(10.95~22.41)	—	10.35(7.61~17.79)	8.55(6.26~14.76)	—
Weighted HLS						
Impaired glucose tolerance	1.38(0.85~2.67)	13.40(8.25~25.77)	10.94(6.71~21.57)	1.78(1.07~4.39)	11.03(6.68~26.47)	4.06(2.45~10.02)
High blood pressure	8.86(7.51~10.78)	8.97(7.61~10.91)	11.33(9.58~13.79)	12.51(10.36~16.47)	6.18(5.12~8.16)	17.11(14.12~22.52)
Hypertriglyceridemia	14.52(12.79~16.88)	13.14(11.58~15.28)	34.67(30.42~40.34)	18.46(14.24~26.69)	15.96(12.29~23.23)	44.82(34.55~63.85)
Low levels of HDL cholesterol	8.94(7.58~11.10)	11.56(9.77~14.30)	—	10.91(8.38~15.99)	8.42(6.49~12.38)	—

Note: SUA、SUA/Cr and UHR were included in the analysis as continuous variables, HLS and weighted HLS were included in the analysis as categorical variables；Models were adjusted for gender, education level, marital status, wealth index, unhealthy eating behaviors, staple foods, acute myocardial infarction, angina, stroke, pulmonary heart disease. HLS: healthy lifestyle score; SUA: serum uric acid; SUA/Cr: serum uric acid to creatinine ratio; UHR: uric acid to HDL cholesterol ratio. "—" means that the relationship between low levels of HDL cholesterol and UHR was not examined since HDL was used to calculate UHR.

Table S9. Mediation impact of SUA, SUA/Cr or UHR on the association between HLS or weighted HLS and components of MetS among the participants without diseases or the imputed sample with multiple imputation

	Excluding the participants with diseases [a] (*n*=7022)			Multiple Imputation [b] (*n*=13072)		
	SUA	SUA/Cr	UHR	SUA	SUA/Cr	UHR
HLS						
Impaired glucose tolerance	2.24(1.32~5.10)	17.56(10.28~39.20)	10.88(6.35~24.93)	7.62(5.17~13.00)	11.14(7.60~19.13)	18.47(12.51~31.97)
High blood pressure	13.78(11.21~17.74)	11.58(9.42~14.89)	18.32(14.84~23.62)	16.01(13.45~19.82)	7.70(6.46~9.51)	21.64(18.15~26.88)
Hypertriglyceridemia	16.93(14.61~20.29)	13.85(11.93~16.64)	35.04(30.28~41.91)	18.47(16.49~21.01)	8.88(7.92~10.11)	35.35(31.61~40.11)
Low levels of HDL cholesterol	11.98(9.48~16.23)	12.30(9.74~16.71)	—	15.14(12.77~18.78)	6.99(5.91~8.66)	—
Weighted HLS						
Impaired glucose tolerance	0.78(0.52~1.36)	18.37(12.32~31.68)	9.38(6.26~16.24)	4.22(3.28~5.73)	10.53(8.25~14.35)	12.22(9.51~16.61)
High blood pressure	11.58(10.06~13.58)	10.07(8.77~11.80)	14.77(12.79~17.32)	9.13(8.31~10.14)	6.21(5.65~6.89)	12.16(11.06~13.52)
Hypertriglyceridemia	16.91(15.07~19.29)	14.73(13.12~16.80)	38.28(34.02~43.84)	16.08(14.85~17.52)	9.84(9.08~10.72)	36.20(33.45~39.50)
Low levels of HDL cholesterol	9.01(7.74~10.71)	10.73(9.22~12.76)	—	9.00(8.14~10.12)	6.11(5.52~6.87)	—

Note: SUA、SUA/Cr and UHR were included in the analysis as continuous variables, HLS and weighted HLS were included in the analysis as categorical variables.

HLS: healthy lifestyle score; SUA: serum uric acid; SUA/Cr: serum uric acid to creatinine ratio; UHR: uric acid to HDL cholesterol ratio. "—" means that the relationship between low levels of HDL cholesterol and UHR was not examined since HDL was used to calculate UHR.

[a] Models were adjusted for gender, age, education level, marital status, wealth index, unhealthy eating behaviors, staple foods.

[b] Models were adjusted for gender, age, education level, marital status, wealth index, unhealthy eating behaviors, staple foods, acute myocardial infarction, angina, stroke, pulmonary heart disease.
